# Quantitative proteomics reveals regulatory differences in the chondrocyte secretome from human medial and lateral femoral condyles in osteoarthritic patients

**DOI:** 10.1186/1477-5956-11-43

**Published:** 2013-10-04

**Authors:** Johan Stenberg, Ulla Rüetschi, Eva Skiöldebrand, Johan Kärrholm, Anders Lindahl

**Affiliations:** 1 Department of Clinical Chemistry and Transfusion Medicine, Institute of Biomedicine, Sahlgrenska Academy at the Sahlgrenska University Hospital, University of Gothenburg, Gothenburg, Sweden; 2 Department of Biomedical Sciences and Veterinary Public Health, Division of Pathology, Pharmacology and Toxicology, Box 7028, SLUS-75007 Uppsala, Sweden; 3 Institute of Clinical Sciences, Department of Orthopaedic Surgery, Sahlgrenska Academy, University of Gothenburg, Gothenburg, Sweden; 4 Clinical Chemistry at Sahlgrenska University Hospital, Bruna Stråket 16, SE-41345 Gothenburg, Sweden

**Keywords:** Secretome, SILAC, Chondrocyte, Osteoarthritis, Proteomics

## Abstract

**Background:**

Osteoarthritis (OA) is a destructive joint disease and there are no known biomarkers available for an early diagnosis. To identify potential disease biomarkers and gain further insight into the disease mechanisms of OA we applied quantitative proteomics with SILAC technology on the secretomes from chondrocytes of OA knees, designated as high Mankin (HM) scored secretome. A quantitative comparison was made between the secretomes of the medial and lateral femur condyle chondrocytes in the same knee since the medial femur condyle is usually more affected in OA than the lateral condyle, which was confirmed by Mankin scoring. The medial/lateral comparison was also made on the secretomes from chondrocytes taken from one individual with no clinically apparent joint-disease, designated as low Mankin (LM) scored secretome.

**Results:**

We identified 825 proteins in the HM secretome and 69 of these showed differential expression when comparing the medial and lateral femoral compartment. The LM scored femoral condyle showed early signs of OA in the medial compartment as assessed by Mankin score. We here report the identification and relative quantification of several proteins of interest for the OA disease mechanism e.g. CYTL1, DMD and STAB1 together with putative early disease markers e.g. TIMP1, PPP2CA and B2M.

**Conclusions:**

The present study reveals differences in protein abundance between medial/lateral femur condyles in OA patients. These regulatory differences expand the knowledge regarding OA disease markers and mechanisms.

## Introduction

Osteoarthritis (OA) is a complex age-related polygenetic progressive degenerative disease of the synovial joint characterized by gradual destruction of the articular cartilage and subchondral bone leading to a loss of joint function. The gradual destruction of the articular cartilage causes joint pain and stiffness, which slowly disables the patient. The OA disease could affect a single joint, several joints or be more generalized. The disease prevalence is higher in women [1-4] and when the knee joint is affected in OA there is a predominant tissue degradation of the medial compartment as compared to the lateral compartment [1,2,5]. Due to the lack of disease modifying treatments the only treatment option for the degenerated joint is eventually a total joint replacement. However, isolated moderate to large knee cartilage defects due to trauma benefit from autologous chondrocytes transplantation procedures [6,7].

The etiology of OA is still elusive although many studies have been performed in order to find pathological mediators and biomarkers [8-11]. When searching for disease processes in OA the whole joint should be considered a diseased organ where the synovium, articular chondrocytes as well as the patellar fat pad contribute with secreted factors, which may have catabolic and/or anabolic effects on articular cartilage [12-15]. Inflammation has been proposed to cause catabolic processes in the joint resulting in cartilage erosion, see Abramson and Attur [16] for a comprehensive review. Signaling pathways as well as autocrine and paracrine mediators have been studied in OA in order to reveal the processes behind the slow regeneration of cartilage [17] and to identify putative drug targets [18]. There have been several genome-wide association studies of osteoarthritis and one of the largest is the arcOGEN study that in its first report from a genome-wide association scan for knee and hip osteoarthritis in 3,177 cases and 4,894 population-based controls was unable to find a genetic linkage to the disease. The conclusion from the study was that “data suggests that osteoarthritis is a highly polygenic disease with multiple risk variants conferring small effects” and that additional larger populations studies are needed [19]. In a second report five genome-wide significant foci were identified [20]. When searching the OMIM database for genes connected to OA GDF5 and FRZB among others are considered potential candidates.

The introduction of global proteomic techniques enables studies of the functional proteome after posttranslational modifications and protein processing, which give a more comprehensive picture in the evaluation of disease hypothesis than solely gene expression analysis. Stable isotope labeling by amino acids in cell culture (SILAC) is a metabolic labeling technique that incorporates nonradioactive isotope labeled amino acids in all newly synthesized proteins [21]. The isotope labeled proteome from one cell culture can be qualitatively and quantitatively compared to another culture condition or cell line cultured with normal amino acids using mass spectrometry (MS). Hence, SILAC combined with MS makes a powerful tool capable of analyzing global proteomic variances. Cultured chondrocytes from OA patients have been studied [22-24] in functional and comparative studies and SILAC approaches in cartilage explants have been tried out but failed to label the total proteome [22].

Our primary hypothesis was that the secretome from culture expanded OA chondrocytes is relevant when compared to the cartilage explants secretome. Secondly we hypothesized that since in OA the medial compartment of the knee joint is more often and more severely affected as compared to the lateral compartment, differences should exist in the secretomes since the cells originate from cartilage with different grades of degeneration. Further, the potential protein abundance differences could expand the knowledge regarding OA disease markers and mechanisms. We here demonstrate that the SILAC technology enables a unique bilateral comparison in the same patient with new interesting results and to our knowledge this is the first time such a comparison has been done.

## Results

### Characterization of cartilage biopsies for monolayer cultures

Total knee replacement biopsies from five OA patients, included in the monolayer culture, were characterized by Mankin score and designated high Mankin scored (HM) patients. The characterization resulted in an average Mankin score for the femoral medial condyle of 9.8 and for the femoral lateral condyle of 4.9 (Table 1). The same characterization was done for the macroscopically healthy cartilage from one individual, which received Mankin score 2.7 for the medial femoral condyle and 0.3 for the lateral femoral condyle (Table 1) and was subsequently designated as a low Mankin score (LM) patient although the medial condyle was slightly affected by an early OA.

**Table 1:** Mankin score of the six monolayer cultured specimens

**Patients**	**Age**	**Mankin score femur medial**	**Mankin score femur lateral**
HM 1	67	10.3	4
HM 2	72	11	5.7
HM 3	72	8.3	4.7
HM 4	69	9	4
HM 5	63	10.3	6.3
LM	70	2.7	0.3

High Mankin Score (HM) Low Mankin Score (LM).

### The relevance of monolayer culture as an experimental model to study the chondrocyte secretome

A previous report has demonstrated the difficulty to incorporate stable isotope labels into proteins in cartilage explant cultures with a relatively small number of identified labeled proteins as a result [22]. This is in agreement with our own results from SILAC labeling of OA explants where stable isotope labeled proteins were present at very low abundance and thus not useful for quantitative analysis (data not shown). We therefore performed stable isotope labeling with amino acids in cell culture (SILAC) of chondrocytes in monolayer cultures. However, in order to evaluate the monolayer culture as an experimental model relevant for studies of differences in the secretome of medial femur condyle and lateral femur condyle positions of the OA knee joint we compared the secretomes in the OA explants and monolayer cultures. For this specific comparison we used the combined secretomes identified from the five monolayer cultures isolated from OA cartilage with high Mankin score and the combined secretomes from the two OA cartilage explants. In the supernatants of OA cartilage explant cultures we identified a total of 344 protein groups at a false discovery rate (FDR) of 1% (Additional file 1: Table S1). An additional filtering for proteins identified with at least two peptides was made, which yielded 320 protein groups. Monolayer cultures of femoral chondrocytes from the five high Mankin scored individuals were grown to full labeling and conditioned with serum-free media for 24 hours. Analysis of the supernatants from the HM monolayer cultures identified a total of 825 protein groups at a FDR of 1% (Additional file 2: Table S2) and additional filtering for proteins identified with at least two peptides yielded 727 protein groups. A comparison of the identified proteins from cartilage explants and monolayer cultured chondrocytes filtered for 1% FDR and identified with at least two peptides showed that 172 proteins were identified in both groups, which represented 54% of the proteins identified in the explant secretome (Additional file 3: Table S3). Polacek et al. used chondrocytes from autologous chondrocyte implantations (ACI) to establish monolayer cultures. Out of the 100 unique proteins identified from their analysis 84 overlapped with our study.

Among the proteins identified in both explant and monolayer cultures were several cartilage extracellular matrix remodeling (ECM) proteins, e.g. cartilage oligomeric matrix protein (COMP), matrix metalloproteinase (MMP)-1, -2, -3 and -10, metalloproteinase inhibitor (TIMP) 1 and 2 and several isoforms of collagen. Genetic association for disease analysis of the 172 proteins common for both monolayer chondrocytes and explant cultures showed the term osteoarthritis to be highly connected to the protein list (P-value ≤ 0.01). Gene ontology analysis for biological process of the 172 common proteins also showed the most enriched terms to be involved in ECM organization and inflammation processes further supporting the hypothesis that the monolayer cultured OA chondrocytes could partly represent the OA secretome (Additional file 4: Table S4, sheet 1). However the well-known loss of phenotype of chondrocytes when subjected to monolayer cultures was also demonstrated by presence of Collagen type I alpha 1 and 2 subunits and absence of Collagen type II subunits in the monolayer secretome (Additional file 2: Table S2).

### Secretome profile of high and low Mankin scored femur chondrocytes

The proteomic analysis of the chondrocyte secretomes from five OA patients undertaking a total knee replacement operation resulted in a comprehensive secretome profile of femoral condyle knee OA chondrocytes. In total, 825 protein groups were identified with a 1% FDR (Additional file 2: Table S2). The corresponding analysis of a low Mankin scored individual resulted in 528 identified protein groups (Additional file 5: Table S5). Gene ontology enrichment analysis of the high Mankin scored secretome regarding cellular compartment and biological process using the DAVID bioinformatics resource showed the highest statistical significance for the GO terms *GO:0044421 extracellular region part* and *GO:0030198 extracellular matrix organization* (Additional file 4: Table S4, sheet 2–3). The corresponding analysis of the low Mankin scored secretome showed high statistical significance for the GO term *GO:0044421 extracellular region part* and interestingly high statistical significance for the biological process terms *GO:0030199 collagen fibril organization* and *GO:0006916 anti-apoptosis* (Additional file 4: Table S4, sheet 4–5).

### Differentially expressed proteins in the femur medial and lateral knee compartments in OA patients

**Figure 1:**
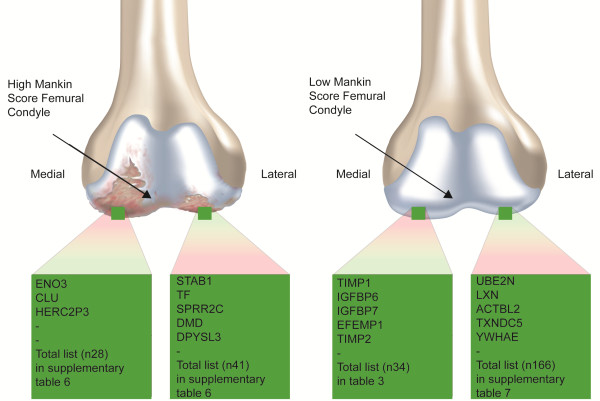
**Schematic view of the femoral condyles.** Proteins listed in green boxes are the enriched secreted proteins from the medial or lateral condyles of the respective HM and LM knee joint.

The secretome analysis from femoral condyle medial and lateral high Mankin chondrocytes within the same knee of five OA patients showed significant differences in the protein amounts of 69 proteins, (Table 2 and Additional file 6: Table S6), among these, 28 protein groups were medially abundant and 41 were laterally abundant (Figure 1). Six proteins were significantly regulated in at least three out of five patients, when comparing the medial and lateral positions. Gene ontology biological process term enrichment analysis of the significantly regulated high Mankin scored secretome showed the highest statistically significant enrichment of the terms *GO:0006954 inflammatory response* and *GO:0009611 response to wounding* (Additional file 4: Table S4, sheet 6). Proteins involved in these processes were Transferrin, Stabilin1, Insulin, Clusterin, S100 calcium binding protein A9, Annexin A1, Desmoplakin, Enolase 3, Complement component 1, r subcomponent, Insulin-like growth factor binding protein 4 and Macrophage migration inhibitory factor. Cellular compartment gene ontology analysis also showed the highest significance for *GO:0005576 extracellular region* (Additional file 4: Table S4, sheet 7).

**Table 2:** Significantly regulated proteins in three out of five high Mankin scored individuals

**Gene names**	**Protein names**	**UniProt**	**SILAC protein ratio**				
			HM. 1	HM. 2	HM. 3	HM. 4	HM. 5
**Significance in 3 of 5 pat.**							
ENO3	Enolase 3	P13929-2	NR	*12.53*	NR	*10.85*	*11.41*
STAB1	Stabilin 1	Q9NY15	*0.18*	*0.05*	*0.02*	0.42	0.05
TF	Serotransferrin	A0PJA6	*0.16*	*0.14*	*0.06*	0.12	0.06
SPRR2C	Small proline-rich protein	Q15515	*0.25*	*0.20*	*0.09*	0.25	NR
DMD	Dystrophin	P11532-1	*0.09*	*0.08*	*0.05*	0.09	0.08
DPYSL3	Collapsin response mediator protein 4 long variant	B3SXQ8	*0.14*	*0.09*	*0.09*	0.11	0.13

Medially abundant protein groups have SILAC ratios >1. Laterally abundant protein groups have SILAC ratios <1. Significant SILAC ratios are in italics. NR= No Ratio.

### Differentially expressed proteins in the femur medial and lateral knee compartments in a low Mankin scored patient

**Figure 2:**
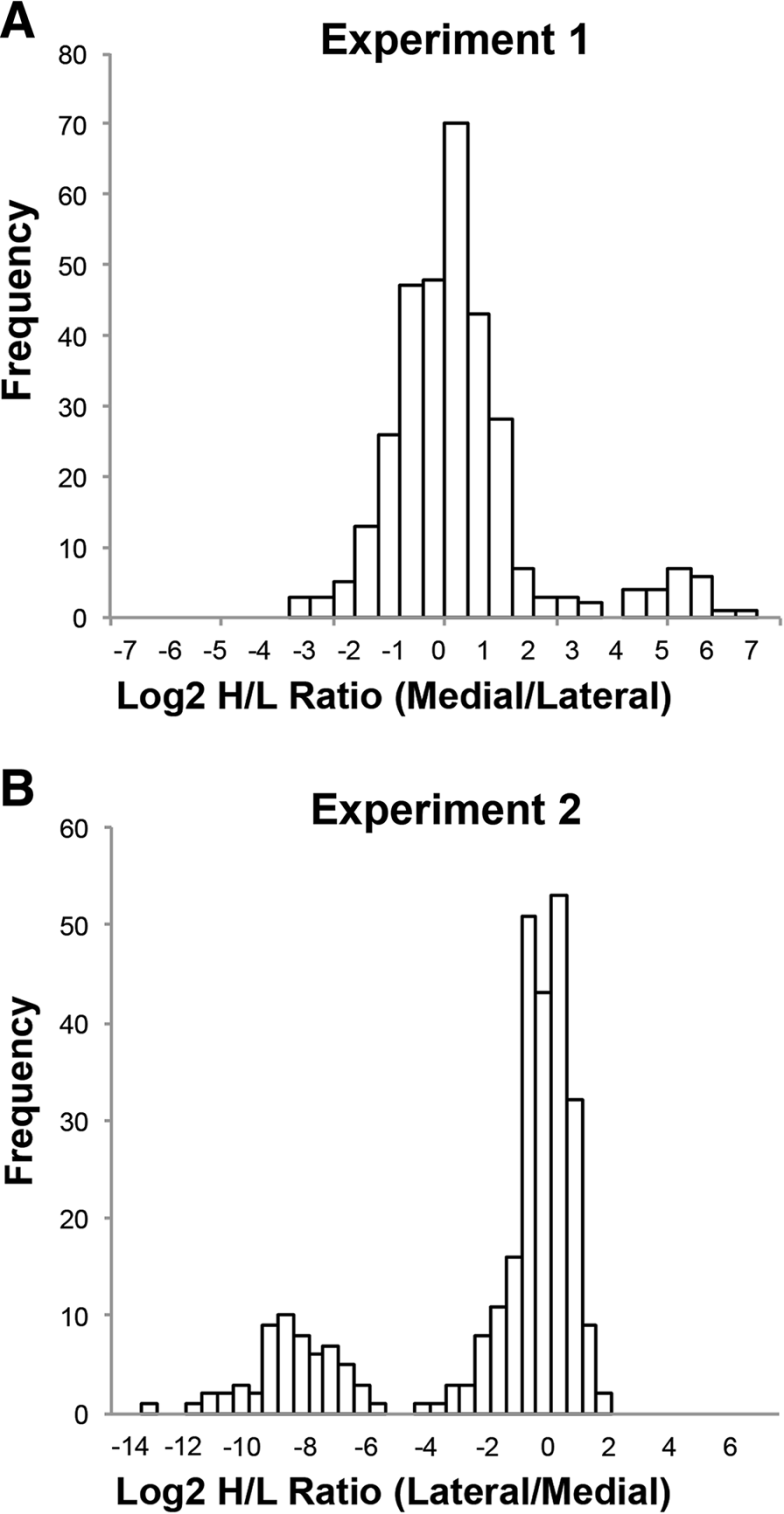
**Bimodal distribution of the Log2 values of the protein H/L ratios from the LM individual.** The Log2 values were normalized to the most frequent value. The bimodal distribution was inverted from the medially labeled sample **(A)** to the laterally labeled sample **(B)**.

Histograms of Log2 values of the protein Heavy/Light (H/L) ratios for the low Mankin scored individual were produced, both labeling experiments resulted in a bimodal distribution (Figure 2). The Significance B could not be calculated since the underlying assumption in this calculation is normal distribution of the data. For these samples the Log2 values were normalized to the most frequent value and proteins with a fold-change above two in either of the experiments were selected. This resulted in 200 identified proteins in the low Mankin scored individual with different levels when comparing the femoral medial and lateral compartments. The medial femoral compartment showed a higher abundance of 34 proteins out of which several are known to affect cartilage homeostasis e.g. TIMP1, TIMP2, SPARC, Col6A1 and Col12A1. Further, Insulin Growth Factor binding protein 6, 7, 3 and 4 were also present at a higher level in the medial compartment of the femoral condyle (Table 3 and Figure 1). Gene ontology analysis of biological process showed statistically significant enrichment for the terms *GO:0001501 skeletal system development*, *GO:0042127 regulation of cell proliferation*, and *GO:0030198 extracellular matrix organization* (Additional file 4: Table S4, sheet 8). In addition, 166 proteins were present at a higher level within the femoral condyle lateral compartment, as compared to the medial compartment, in the low Mankin scored individual (Table 4, Additional file 7: Table S7 and Figure 1). The laterally abundant protein groups showed significant term enrichment results in gene ontology analysis for *GO:0030036 actin cytoskeleton organization* e.g. Destrin, *GO:0006006 glucose metabolic process* e.g. Enolase 1 and *GO:0006916 anti-apoptosis* e.g. Annexin a1 (Additional file 4: Table S4, sheet 9). Interestingly, the femoral medial and lateral protein SILAC ratios seen in the LM scored individual were lost in the HM patients, i.e. the SILAC ratio between the medial and lateral femur condyle compartments were close to one (Tables 3, 4 and Additional file 7: Table S7). Thus, the quantitative difference in protein secretion between the medial and lateral compartments found in the low Mankin scored individual is normalized in the medial and lateral compartments in the high Mankin scored individuals.

**Table 3:** Enriched protein groups in the medial femur condyle of the low Mankin individual and SILAC ratios in all individuals

**Gene names**	**Protein names**	**UniProt**	**SILAC protein ratio**		**SILAC protein ratio**				
			LM I	LM II	HM. 1	HM. 2	HM. 3	HM. 4	HM. 5
TIMP1*	Tissue inhibitor of metalloproteinases 1	P01033	95.7692	0.0027	1.5998	1.1847	1.3414	0.4638	0.8376
IGFBP6*	Insulin-like growth factor binding protein 6	P24592	68.0973	0.0018	NR	0.1656	NR	0.1418	1.0172
IGFBP7*	Insulin-like growth factor-binding protein 7	Q16270	56.9646	0.0027	1.4450	1.4334	2.8779	1.6507	1.4007
EFEMP1*	EGF-containing fibulin-like ECM protein 1	Q12805	55.6707	0.0018	1.1036	0.2390	2.2981	0.3037	0.2289
TIMP2*	Tissue inhibitor of metalloproteinases 2	P16035	55.6099	0.0016	1.6216	1.3963	NR	0.9244	1.0870
FSTL1*	Follistatin-like protein 1	Q12841	50.4631	0.0036	1.7105	1.1239	3.4944	1.1685	1.0447
CST3*	Cystatin-3	P01034	48.8661	0.0038	1.5492	0.5319	NR	0.9870	1.1611
IGFBP3	Insulin-like growth factor-binding protein 3	P17936	47.2340	0.0019	1.2820	0.3277	NR	0.5362	0.8611
CHI3L1*	Cartilage glycoprotein 39	P36222	45.1381	0.0040	0.7167	0.8409	3.2179	0.4490	0.3307
LUM*	Lumican	P51884	44.3039	0.0025	1.4396	1.1753	1.7528	0.9746	0.6962
SERPINE2*	Serpin E2	B4DIF2	39.5572	0.0062	0.7233	2.3149	NR	2.2748	NR
IGFBP4*	Insulin-like growth factor-binding protein 4	P22692	39.1861	0.0022	0.3036	0.2409	1.4139	0.2510	0.1026
SPARC	Osteonectin	P09486	36.7189	0.0050	1.1554	0.5516	3.3970	1.1862	0.4348
CTSB*	Cathepsin B	P07858	36.2281	0.0045	0.8972	1.7551	NR	0.8085	0.7847
PCOLCE*	Procollagen C-endopeptidase enhancer 1	Q15113	33.0022	0.0021	1.1413	1.6081	NR	0.6564	0.8188
QSOX1*	Quiescin Q6 Sulfhydryl oxidase 1	O00391	30.8917	0.0028	0.3646	0.2358	NR	0.3496	0.1460
SERPINE1*	Serpin E1	P05121	24.9099	0.0107	1.9987	0.3301	1.1686	0.8524	0.8781
CYTL1*	Cytokine-like protein 1	Q9NRR1	23.5861	0.0038	NR	NR	NR	0.3065	0.1061
FBN1	Fibrillin-1	P35555	23.5613	0.0042	1.5900	0.7567	1.2034	0.7985	1.1273
COL12A1*	Collagen alpha-1(XII) chain	Q99715	22.5922	0.0088	0.7853	0.7624	1.2400	0.5582	0.7052
B2M*	Beta-2-microglobulin	P61769	20.8405	0.0136	1.2302	1.8824	2.5573	1.0522	0.9875
COL6A1*	Collagen alpha-1(VI) chain	P12109	17.4001	0.0076	1.1075	1.0954	NR	0.9453	1.0361
S100A8*	S100 calcium-binding protein A8	P05109	10.4904	0.0016	NR	0.2692	0.4050	0.2882	NR
COL1A2	Collagen alpha-2(I) chain	P08123	6.9371	0.0079	0.7605	0.5581	NR	0.3964	0.9798
FN1	Fibronectin 1	Q14328	6.5113	0.0077	NR	NR	NR	0.6257	NR
COL1A1*	Collagen alpha-1(I) chain	P02452	5.9307	0.0219	0.9983	1.0441	0.9875	0.9924	1.0064
TPD52L2	Tumor protein D52-like 2	Q5JWU6	3.2973	0.5148	1.2657	NR	NR	NR	NR
CFB	Complement factor B	Q53F89	3.1730	0.0029	NR	NR	NR	0.6802	NR
CLEC3B*	C-type lectin domain family 3 member B	P05452	2.6487	0.0037	NR	2.4076	NR	1.0038	0.7486
TUBB	Tubulin beta chain	P07437	2.4448	0.5533	1.1187	NR	1.1792	NR	NR
CALU	Calumenin	B3KPG9	2.2541	0.1224	1.1768	1.2613	1.3553	1.6319	1.4068
UGDH	UDP-glucose 6-dehydrogenase	O60701	2.1286	0.4052	1.1356	NR	NR	NR	NR
DSP*	Desmoplakin	P15924	2.0360	0.0050	0.2379	0.2814	0.1133	0.1970	NR
IDH1	Isocitrate dehydrogenase 1 (NADP+), soluble	O75874	1.9970	0.3614	1.0508	NR	NR	NR	NR

LM I = Low Mankin scored individual experiment one. LM II = Low Mankin scored individual experiment two. HM.1-5 = High Mankin scored individual 1–5. NR = No Ratio.

* = Proteins identified both in the explant and monolayer secretomes.

**Table 4:** The most up-regulated proteins in the LM femur lateral secretome

**Gene names**	**Protein names**	**UniProt**	**SILAC protein ratio**		**SILAC protein ratio**				
			LM I	LM II	HM. 1	HM. 2	HM. 3	HM. 4	HM. 5
UBE2N	Ubiquitin-conjugating enzyme E2 N	P61088	0.0124	9.5421	0.9613	0.6917	NR	NR	0.8378
LXN	Latexin	Q9BS40	0.0134	5.9971	NR	0.6758	NR	NR	0.5899
ACTBL2*	Beta-actin-like protein 2	Q562R1	0.0233	4.9112	0.9809	0.5849	0.7814	0.2662	0.5782
TXNDC5	Thioredoxin domain-containing protein 5	Q8NBS9	0.0262	11.4810	0.8763	0.9413	0.8426	0.7740	0.6789
YWHAE	14-3-3 protein epsilon	P62258	0.0352	12.7140	1.1072	0.9430	1.0612	0.9204	0.8249
CA2	Carbonic anhydrase 2	P00918	0.0353	11.8720	0.7900	0.8925	1.3874	0.9696	0.5556
FHL1	Four and a half LIM domains protein 1	Q13642-1	0.0356	18.3770	1.0419	0.5525	0.7408	0.9364	1.1789
PFN1	Profilin I	P07737	0.0381	10.4650	1.0302	0.8745	0.8038	0.9245	1.0736
PGAM1*	Phosphoglycerate mutase 1	P18669	0.0388	10.9160	0.9279	0.7419	0.6064	1.1168	0.9556
TPI	Triosephosphate isomerase	Q53HE2	0.0424	11.0430	1.0331	1.0640	1.1490	1.0830	1.1780
RSU1	Ras suppressor protein 1	Q15404	0.0428	8.9161	1.0096	0.6922	0.6630	NR	0.8345
GSTO1	Glutathione S-transferase omega-1	P78417	0.0430	23.5890	0.9591	0.8911	NR	NR	NR
AKR1B1	Aldehyde reductase	P15121	0.0439	21.0670	0.7693	1.0281	1.0159	NR	0.8704
STMN1	Stathmin	B7Z8N4	0.0452	13.8150	0.9782	0.6731	1.3752	0.6255	0.8742
YWHAB	14-3-3 protein beta/alpha	P31946-2	0.0456	48.1920	1.0435	0.9433	0.8530	0.9862	0.8862
ALDOA	Fructose-bisphosphate aldolase A	P04075	0.0458	11.5280	0.9721	0.6382	0.7641	0.9858	1.0069
YWHAB	14-3-3 protein beta/alpha	P31946-1	0.0465	14.3180	NR	0.9594	NR	NR	NR
MDH1	Malate dehydrogenase, cytoplasmic	P40925	0.0465	14.4410	1.0407	0.9923	1.0104	1.0328	0.8833
FTL	Ferritin light chain	P02792	0.0488	24.5770	0.7289	0.6701	NR	NR	0.5221
YWHAZ*	14-3-3 protein zeta/delta	P63104	0.0498	18.0160	1.0942	0.9132	1.0023	1.0695	0.9371
EIF5A	Eukaryotic translation initiation factor 5A-1	P63241-2	0.0550	16.9460	1.0730	0.8086	NR	NR	0.9224
ARHGDIA	Rho GDP-dissociation inhibitor 1	P52565	0.0556	14.7570	0.8920	0.6763	NR	0.9223	0.8761
LDHB	L-lactate dehydrogenase B chain	P07195	0.0582	13.1470	0.9911	0.7294	0.7403	0.8234	0.8016
AKAP12	A-kinase anchor protein 12	Q02952-1	0.1125	22.0240	NR	NR	NR	NR	NR
SCRN1	Secernin-1	Q12765	0.1244	11.0260	NR	NR	NR	NR	NR
ANXA1*	Annexin A1	P04083	0.1407	99.8340	1.8046	0.8261	1.1868	NR	1.2625
EPB41L2	Band 4,1-like protein 2	O43491	0.1462	11.4150	NR	NR	NR	NR	NR
FABP3	Fatty acid-binding protein 3	P05413	0.1541	23.3740	NR	NR	NR	NR	NR
ANXA5*	Annexin A5	P08758	0.1546	6.0555	1.4520	NR	1.1515	NR	NR
S100A11*	Protein S100-A11	P31949	0.1830	7.7868	1.1976	0.8693	0.8680	0.9675	1.0495
CDV3	Protein CDV3 homolog	Q9UKY7-1	0.2226	16.6240	NR	NR	NR	NR	NR

Total list is available in the Additional file 7: Table S7.

LM I = Low Mankin scored individual experiment one. LM II = Low Mankin scored individual experiment two. HM.1-5 = High Mankin scored individual 1–5. NR = No Ratio.

* = Proteins identified both in the explant and monolayer secretomes.

### Verification of MS results by ELISA

**Figure 3:**
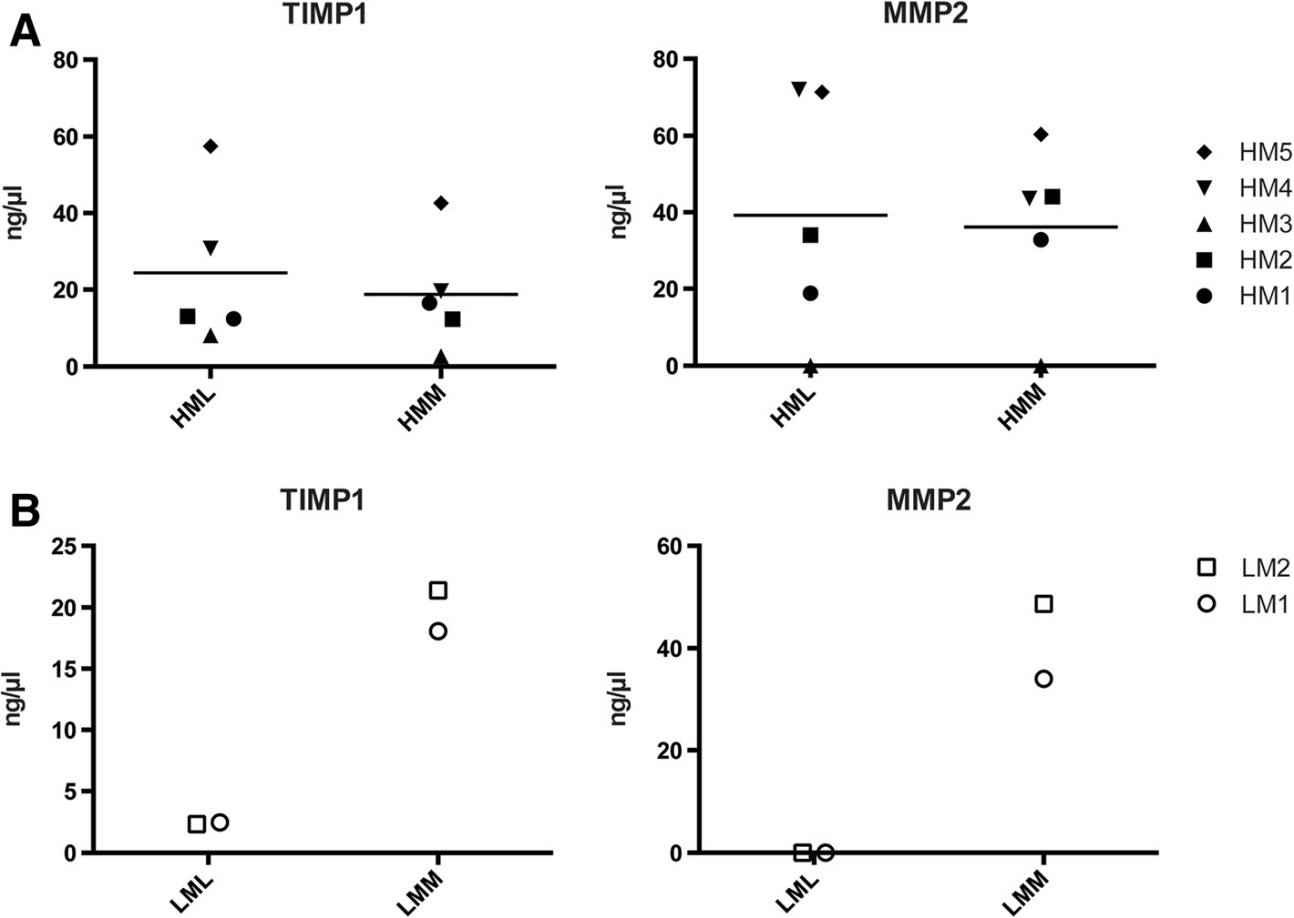
**ELISA of MMP2 and its inhibitor TIMP1 levels in the HM individuals (A) and the LM individual (B).** The two measuring points in the LML and LMM group represent the two individually labeled culturing experiments of the low Mankin scored individual. High Mankin Scored Lateral Chondrocytes (HML), High Mankin Scored Medial Chondrocytes (HMM), Low Mankin Scored Lateral Chondrocytes (LML) and Low Mankin Scored Medial Chondrocytes (LMM).

The MS data of two of the most prominent ECM remodeling proteins, i.e. TIMP1 and MMP2, were verified with ELISA. The ELISA confirmed that the levels of MMP2 and the known MMP inhibitor TIMP1 were approximately equal between the medial and lateral compartments in the HM individuals. Further the ELISA also verified that the SILAC ratio values for the LM individual showed a medial abundance of TIMP1 (Figure 3). MMP2 was identified in the secretome from the LM individual but a SILAC ratio could not be generated. Further analysis of the MS data showed that the identified MMP2 peptides from the LM individual included the stable isotope labeled amino acids indicating that the MMP2 was mainly generated from the medial compartment of the LM femoral condyle. ELISA confirmed this observation since the lateral chondrocytes from the LM individual did not secrete any MMP2 at levels detectable by ELISA while the medial chondrocytes secreted MMP2 in amounts within the levels from the HM chondrocytes (Figure 3). Taken together ELISA confirmed the MS results for TIMP1 and MMP2 in all samples.

### Proteins uniquely identified in low Mankin scored chondrocytes

We finally addressed the possibility of identifying unique proteins only secreted in the LM scored secretome and not in the secretome of the five HM scored individuals. Indeed, we found 25 unique proteins in the LM scored individual where 5 were found in both LM labeled experiments (EPB41L2, AKAP12, FABP3, SCRN-1, CDV3) (Additional file 8: Table S8).

## Discussion

This study was designed to quantitatively compare, using SILAC technology, the secretome from medial and lateral femoral condyle chondrocytes obtained from OA patients undergoing total knee replacement and from an aged and sex matched individual with no clinical joint disease as a hypothesis generating approach towards a better understanding of OA. The LM sample became highly interesting since the LM medial side data suggested an early stage OA compared to the non-affected LM lateral side, such early stage OA samples are very rare in literature and have never been examined with the SILAC-technology before.

Results from the first studies that applied the SILAC technology on articular cartilage were qualitative identifying newly synthesized proteins [22,25]. Later studies performed by Calamia et al. were both qualitative and quantitative [23,24] but to our knowledge our study is the first that qualitatively and quantitatively compares medial and lateral compartments with different grades of cartilage degeneration within the same knee.

Previous attempts to label the explant secretome by the SILAC-technique has proven difficult in articular cartilage and our own attempt to label the explant secretome gave similar results (data not shown) as the study by Polacek et al. where only 25 to 30% of the explant secretome was labeled possibly due to the slow cell cycle of chondrocytes within the cartilage ECM or interference from the ECM itself [22]. This is a methodological drawback since quantitative evaluation of SILAC experiments is only interpretable if the labeled proteome is fully saturated with the stable isotope. Therefore we widened our study with a first aim to address the hypothesis whether the monolayer chondrocyte culture secretome could mirror the secretome from native explants and thus partly represent the OA secretome. The present study demonstrates that a substantial proportion of the detected proteins secreted from explants (54%) were also detected among the secreted proteins from monolayer cultures and that several proteins important for ECM remodeling and reflecting OA disease were found in the common secretome. Our conclusion is that although monolayer culture has its limitations in respect of phenotype change e.g. regarding Collagen type II the identified monolayer secretome indicate an association with OA. However, to validate the findings in the present study there is an absolute need for experimental improvements in future quantitative studies of the explant secretome. Such improvements could involve modifications of the incubation protocols and use of thinner explant slices. An alternative labeling method that could have been used is iTRAQ/TMT but it does not solve the problem with quantitative analysis of newly synthesized proteins versus proteins present within the cartilage extracellular matrix.

We studied the secreted proteins from chondrocytes as they have particular importance in tissue maintenance and regeneration as well as their putative usage as biomarkers. Further, it is important to study the secreted proteins since only approximately 7% of the proteins found in the intracellular proteome of chondrocytes may have a direct role in cartilage ECM synthesis/turnover [23].

The cartilage specimens used in this study were scored according to the Mankin scoring system [26], a well accepted and validated score for histological grading of cartilage histology [27]. The sample obtained from the patient with no history of joint disease had a medial Mankin score of 2.7 and a lateral Mankin score of 0.3. The different scores indicated that although the LM scored patient was asymptomatic from any joint disease the medial compartment was weakly affected and could hypothetically be regarded as a mild early stage of OA. The changes in protein secretion between the medial and lateral side are thus interesting as potential markers of early OA. The single LM scored sample gave no possibility to draw any statistical conclusions regarding the proteomic state within normal knee joints. However, the results from the LM scored specimen are well experimentally documented by repeated independent cultures from the medial and lateral compartments in both labeled and unlabeled medium, respectively. In this way we could analyze the secretome twice with inverted labeling conditions, which gave strong technical data that supported the validity of the results.

In the HM scored OA patients we identified 825 proteins and 69 of these showed to have a significantly changed abundance when comparing the femoral medial and lateral compartments.

The 69 significantly altered proteins were further analyzed in the DAVID where the proteins were significantly annotated to the terms *inflammatory response* and *response to wounding*, which correlates with OA disease parameters. Enolase 3, Stabilin 1 and Transferrin were among the six proteins that were significantly regulated in three of five HM individuals and interestingly annotated as response to wounding (Table 2). Enolase 3 was medially elevated and Stabilin 1 and Transferrin were laterally elevated. Interestingly, Stabilin 1 has been shown to be involved in macrophage mediated clearance of SPARC, a protein which is thought to be involved in mineralization of bone and is known to be up-regulated in OA [28-31]. In this study Stabilin 1 is less abundant in the HM femoral medial compartment, which could result in a reduced localized SPARC clearance contributing to the medial compartment OA pathogenesis. Transferrin is known to be synthesized by hypertrophic chondrocytes and is thought to be a chemotactic factor promoting migration of endothelial cells [32]. In OA cartilage this could be a reparative response that is down-regulated in the more damaged femur medial compartment. The remaining three significantly regulated proteins were not included in a single significantly enriched GO term; dystrophin (DMD); small proline rich protein (SPRR2C) and Collapsin response mediator protein 4 long variant (DPYSL3), however SPRR2C was included in the significantly enriched term *GO:0009913 epidermal cell differentiation*. SPRR2C and DPYSL3 have important cellular functions but no known function in cartilage [33,34]. Interestingly, DMD deficient female mice that had the opportunity to voluntary exercise were affected by a significantly reduced cartilage thickness and cartilage tissue area on the proximal femur head compared to wild type control [35]. The present study shows DMD levels to be significantly reduced in the femoral medial more damaged compartment compared to the lateral more intact compartment of the OA knee as evaluated by Mankin score. Thus DMD may have an ECM preserving role in hyaline cartilage and interestingly DMD was annotated as *GO:0042592 homeostatic process*. To our knowledge this study is the first to report the involvement of DMD in human OA. Taken together, the MS results from the HM scored individuals include a comprehensive secretome of the OA phenotype together with proteins that may mark the OA progression between the affected lateral compartment to the heavily affected medial compartment.

We found several interesting proteins in the LM scored individual to be elevated with a fold change over 2 in the femoral medial compartment where Cystatin-3, Timp1 and B2M were among the most medially elevated proteins. Cystatin-3 is a cysteine protease inhibitor, which is known to modulate the effect of the cysteine proteinase Cathepsin B [36]. Cathepsin B was also elevated in the femoral medial compartment of the healthy individual, which may indicate an early state of OA in the medial compartment supported by the facts that elevated Cathepsin B levels has been found in OA specimens [37]. This is in accordance with the higher Mankin score in the femur medial compartment of the LM scored individual. The effect of Cathepsin B may however be balanced by the elevated levels of Cystatin-3.

TIMP1 and TIMP2 are known endogenous inhibitors of MMPs, which makes them key factors in the cartilage ECM homeostasis. The elevated TIMP1 and TIMP2 levels found in the LM score individual in the femoral medial chondrocyte secretome may be a reactive mechanism in response to an early OA as the medial condyle had a higher Mankin score. This was further supported by the identification of MMP2 in the MS-analysis, even though the levels in the lateral compartment were not sufficient for determination of a SILAC-ratio. Furthermore, ELISA confirmed MMP2 to be only present in the medial secretome of the LM individual. The C-terminal fragments of Procollagen C-endopeptidase enhancer 1 (PCPE) have been shown to inhibit MMP2 [38]. PCPE, also known as PCOLCE, levels were elevated in the LM femoral medial chondrocyte secretome, which is the same abundance pattern as MMP2 in this individual. PCOLCE was further expressed in all MMP2 expressing HM scored chondrocytes but was absent in the secretome from the HM individual nr 3 where no MMP2 was detected by either MS or ELISA. Taken together these results indicate that MMP2 and PCOLCE levels are connected to each other possibly due to a protective feedback loop. B2M has previously been proposed to be involved in OA pathogenesis as B2M is expressed in OA cartilage and may influence chondrocyte gene expression as well as inhibit proliferation of chondrocytes [39]. The medially up-regulated B2M levels found in the LM scored individual could potentially balance the proliferative stress signals that may be initiated by normal joint usage and/or be the early response to a low grade OA and thus a potential early marker of OA. Our group has previously reported that chondrocytes from different locations within the knee joint have different production of proteoglycans and collagens together with different chondrogenic potential in vitro [40]. Interestingly, the present study demonstrates that the differences in abundance of the medially/laterally regulated proteins in the LM scored individual were abolished in OA patients indicating that a homeostatic balance within the LM scored knee may be impaired in the HM scored OA knee, however this hypothesis must be further evaluated in the future.

The low TIMP1 and MMP2 ELISA values for the HM3 patient (with the lowest Mankin score within the HM group) could indicate an intermediate disease stage for this patient, which is further supported by the similarities in SILAC ratios with the LM scored patient. Such SILAC ratios were seen for IGFBP7, EFEMP1, FSTL1, CHI3L1, IGFBP4, SPARC and B2M. EFEMP1 or formerly Fibulin-3 was demonstrated to have a negative regulation on chondrocyte differentiation and was proposed as a diagnostic biomarker for OA [41,42]. FSTL1 has been demonstrated to be correlated to the severity of OA disease as well as CHI3L1 [43-45]. SPARC and B2M are correlated to OA while the IGFBP7 has not been correlated to OA previously [30,39,46]. IGFBP4 is suggested to inhibit the canonical Wnt signaling pathway, which makes elevated levels of IGFBP4 on the medial side in the LM scored specimen an interesting finding as stimulated Wnt signaling is known to be involved in OA pathogenesis [47-50]. This SILAC ratio pattern may indicate an OA initiation in the medial femur condyle of the LM scored individual and a counteracting reparative process, which has balanced cartilage homeostasis in this aged and sex matched individual who never developed clinical signs of OA. When the lateral side becomes more affected by the OA, as in the HM3 patient, the ratio becomes smaller towards a ratio close to one seen in the highest Mankin scored patients.

One of the medially abundant proteins in the LM individual is the Cytokine like protein 1 (CYTL1) which is only found in two HM patients with an elevated level on the femur lateral side. CYTL1 is a novel autocrine regulatory factor that regulates chondrogenesis of mouse mesenchymal cells [51]. In a recent knockout study deletion of the CYTL1 gene did not affect chondrogenesis or cartilage development. However, CYTL1 knockout mice were more sensitive to osteoarthritic cartilage destruction and expression levels of CYTL1 were markedly decreased in OA cartilage of humans and experimental mice [52]. Our SILAC data thus support the hypothesis that CYTL1 is required for the maintenance of cartilage homeostasis, and loss of CYTL1 function is associated with OA cartilage destruction.

SILAC ratios showing laterally abundant proteins in the LM individual were also analyzed and showed several proteins designated as anti-apoptotic (e.g. Annexin 1 and 5). Chondrocyte derived S100A8 and S100A9 have a potential role in inflammatory arthritis in initiating early cartilage degradation by up-regulating MMPs and aggrecanases [53], partly supported by our results with laterally abundant S100A11 and medially abundant S100A8 in the LM scored individual.

Finally we analyzed the MS data for unique proteins in the LM scored secretome that were not found in any of the high Mankin scored patients. Interestingly we found 25 unique proteins in the LM scored secretome and all proteins found were elevated on the lateral femoral side except for the coatomer subunit zeta-1. Five of the proteins were also found in the inversely labeled LM scored experiment i.e. EPB41L2, AKAP12, FABP3, SCRN-1, CDV3 and are thus of high relevance and certainty. The A-kinase anchor protein 12 (AKAP12) has a pivotal role in regulation of cellular adhesion dynamics and is also a putative inhibitor of angiogenesis through down-regulation of MMP9 expression [54]. MMP9 inhibition through AKAP12 may thus suppress the typical hallmarks of OA in the LM scored individual i.e. ECM degradation and angiogenesis in the cartilage. Secernin-1 (SCRN-1) regulates exocytosis in mast cells and has recently been proposed as a prognostic marker in synovial sarcoma where high expression is associated with a better prognosis possibly due to a higher grade of differentiation [55,56]. Thus, high SCRN-1 levels could mark a differentiated cartilage, and in the case of the LM individual mark a more dedifferentiated OA-like state in the medial compartment as the levels were medially lowered. However, these conclusions may be tissue specific as SCRN-1 expression has also been proposed as a prognostic marker in colorectal cancer where elevated expression was associated with a worse prognosis [57]. The FABP3 protein is related to regulation of apoptosis where it both promotes and inhibits apoptosis, which may be of interest in further studies regarding its role in OA [58-60]. Among proteins that only generated a protein SILAC ratio in one LM experiment PPP2CA is the catalytic subunit of protein phosphatase 2A (PPP2A). Inhibition of PPP2A in TGFbeta stimulated normal human chondrocytes has an anti apoptotic effect [61]. Inhibition of PPP2A is also suggested to have a chondrogenic effect through the protein kinase A signaling pathway in experiments with chicken chondrocytes in vitro [62,63]. Our results indicate that PPP2CA levels are lowered in the medial secretome of the LM individual and not present in the HM secretomes. This could indicate that loss of PPP2CA is an early OA marker in the medial compartment of the LM individual that is completely lost in the end stage of the disease. However, the actual role of PPP2CA in OA needs to be further investigated.

## Conclusions

We have demonstrated that proteins detected in the monolayer cultured chondrocyte secretome partly mirrors the proteins detected in the explant secretome and allows for quantitative studies. By applying the SILAC technology we demonstrated that the relative abundance of proteins significantly differed between chondrocytes isolated from the lateral and medial femoral condyle of OA patients (represented by Enolase 3, Stabilin 1, Transferrin, DMD SPRR2C and DPYSL3). Furthermore, in the LM scored chondrocyte secretome there were several proteins with a high SILAC ratio between the femur medial and lateral compartment e.g. MMP2 and TIMP1, that in combination with the Mankin scores may suggest that the LM medial femoral condyle secretome represents an early OA. Such unique data has to our knowledge never been published. Thus the differently expressed proteins could be potential markers of early OA degradation e.g. PPP2CA, IGFBP7, EFEMP1, FSTL1, CHI3L1, IGFBP4, SPARC and B2M. The high SILAC ratios in LM scored chondrocyte secretomes are lost in the HM scored chondrocyte secretomes, which indicates a shift in protein expression as the HM lateral femur compartment becomes more affected by the upgraded OA. Also, our data confirm experimental findings in mice regarding the role of DMD and CYTL1 in OA. There are also additional unique proteins for the LM scored patient that could represent healthy cartilage or early OA and could have potential for future functional studies e.g. new drug targets. For highlighted findings see list of proteins highlighted in the discussion.

### List of proteins highlighted in the discussion

Gene name

Markers of early OA

PPP2CA

IGFBP7

EFEMP1

FSTL1

CHI3L1

IGFBP4

SPARC

B2M

Regulators of cartilage regeneration and differentiation

B2M

TF

CYTL1

IGFBP4

Regulators of extra cellular matrix homeostasis

STAB1

DMD

TIMP1

TIMP2

MMP2

CST3

PCOLCE

S100a8

## Material and methods

### Patient data and cartilage harvest

Human cartilage material used in this study was acquired from seven female total knee replacement cases suffering from severe OA and one female patient with macroscopically healthy knee cartilage. From two individuals, 73 and 82 years old undertaking total knee replacement, cartilage explants were obtained from the medial and lateral femur condyles. Chondrocytes for monolayer cultures were separately extracted from the medial and lateral femur condyle of the remaining five total knee replacement patients, 63, 67, 69 and two 72 years of age. The sixth monolayer culture was performed from macroscopically healthy cartilage biopsies taken from a patient 70 years of age undertaking leg amputation due to sarcoma with a macroscopically healthy knee cartilage. All samples were provided by the orthopedic surgeon department at Sahlgrenska University Hospital in Gothenburg with patient informed consent. Cartilage donations were approved by the ethical committee at the Sahlgrenska Academy at the University of Gothenburg, Gothenburg, Sweden.

### Biopsy preparation for Mankin classification

Biopsies were isolated and a section of each biopsy was fixated in Histofix (Histolab products, Gothenburg, Sweden) for 24 hours, dehydrated with serial baths of increasing ethanol concentrations and embedded in paraffin. The paraffin embedded biopsies were cut into 5 μm sections onto microscope glass slides (Superfrost Plus, Menzel-Gläser, Germany), deparaffinized, Saffranin O stained and examined in a light microscope (Nikon, Japan). The biopsies were subsequently classified according to the modified Mankin score where the inspection of the tidemark has been excluded [26]. Mankin score is presented as mean out of three individual blinded scoring sessions performed by three experienced technicians. The OA specimens achieved higher Mankin scores and are in the following text accordingly denoted high Mankin (HM) patients while the unaffected cartilage specimen is denoted Low Mankin scored (LM).

### Cartilage explant culture

Cartilage explants from two individuals were used for investigation of the explant secretome. Individual specimens were collected from the medial and lateral femur condyles respectively. Samples were cut into small pieces (approximately 2 mm^3^) and cultured for SILAC analysis. Femoral medial explants were incubated in SILAC DMEM/F12 medium containing 0.1 mg/mL of stable isotope labeled (heavy) arginine and lysine (U-^13^C_6_-Arginine and U-^13^C_6_-Lysine, Invitrogen) while femoral lateral explants were incubated in SILAC DMEM/F12 supplemented with 0.1 mg/mL normal (light) arginine and lysine (Invitrogen). The explant medium was changed daily for 10 days. Equal volumes (2 mL) of the conditioned explant culture medium from stable isotope labeled and unlabeled cultures were mixed prior to proteomic analysis. Duplicate samples of medium collected at day 2 and 10 from each patient were analyzed resulting in eight data-files representing the explant secretome.

### Stable isotope labeling of chondrocytes in monolayer culture

Chondrocytes were separately extracted, as described earlier [6], from the medial and lateral femur condyle of five HM-scored patients affected with severe OA and from one LM-scored individual with macroscopically healthy knee cartilage. The extracted chondrocytes were seeded, at 16 × 10^3^ cells/cm^2^, in SILAC DMEM/F12 medium (HM 1–5 and LM Experiment I). For the six chondrocyte cultures from the medial femur condyle the SILAC DMEM/F12 medium contained 0.1 mg/mL of stable isotope labeled arginine and lysine (U-^13^C_6_-Arginine and U-^13^C_6_-Lysine, Invitrogen) and for the six chondrocyte cultures from the lateral femur condyle the SILAC DMEM/F12 medium contained 0.1 mg/mL of normal arginine and lysine (Invitrogen). In addition, the LM scored cells were also cultured with the inverted labels (LM experiment II). Medium was changed every third day. All cultures were conducted in 37°C, 95% humidity and 5% CO_2_.

### Protein enrichment and digestion

**Figure 4:**
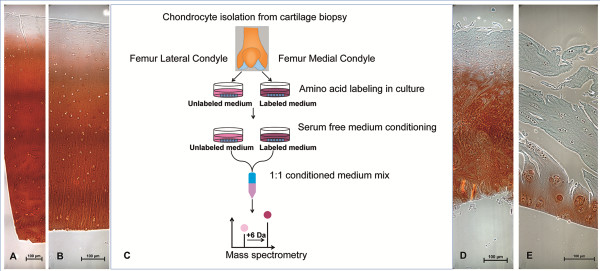
**Representative biopsy images and experimental line-up.** Saffranin O staining of the LM lateral femur condyle **(A)**, LM medial femur condyle **(B)**, representative HM lateral femur condyle **(D)** and representative HM medial femoral condyle **(E)**. Schematic view of the experimental procedure where chondrocytes were extracted from lateral and medial femoral condyle of the same knee joint, labeled in culture, incubated for medium conditioning and analyzed with MS **(C)**.

Supernatants from the cartilage explant cultures were collected after 2 and 10 days, respectively. Two aliquots of 2 mL from each time point were concentrated to 50 μL and the proteins were separated on SDS-PAGE gels as described below. After monolayer expansion of chondrocytes in SILAC-labeled (^13^C_6_-Lysine and ^13^C_6_-Arginine) or SILAC-unlabeled (^12^C_6_-Lysine and ^12^C_6_-Arginine) media for 5–6 cell doublings cells were conditioned in serum-free medium for 24 hours and subsequently the serum free conditioned media were collected and processed prior to MS-analysis. Equal volumes (2 mL) of the conditioned cell culture medium from SILAC-labeled and unlabeled cultures were mixed and the volume was reduced from 4 mL to approximately 50 μL on the Amicon Ultra-2 centrifugal concentrators (Millpore) with a molecular cut-off of 3,000 Da. The concentrated protein secretome in the retentate was separated by denaturing one-dimensional polyacrylamide gel electrophoresis (1-D SDS-PAGE). The concentrated protein solution (25 μL) was supplemented with NuPage LDS-sample buffer (Invitrogen) and dithriothreitol to a final concentration of 50 mM. Proteins were reduced and denatured by heating at 70°C for 10 minutes and loaded onto 4-12% Bis-Tris (NuPAGE® Novex) precast polyacrylamide gels, post-separation, proteins were visualized by Coomassie colloidal blue staining (Invitrogen). For in-gel trypsin digestion each gel lane was divided into 15 equally sized gel slices and subjected to automated trypsin digestion on the BioMek 2000 workstation equipped with a vacuum manifold. 96-well plates supplemented with a 7 μL volume of C18 reversed phase chromatographic resin (Lab-in-a-Plate Flow-Thru Plate, Glygen) were used for vacuum filtration and sample clean-up. The work-flow essentially followed the protocol previously described [64] except that the peptide extraction was performed twice with 0.2% trifluoroacetic acid (TFA) to allow for peptide binding to the C18 resin of the filter plates. Finally, peptides were eluted in two times 40 μL of 60% acetonitrile in 0.1% TFA and the eluted fractions were evaporated to dryness in a speedvac centrifuge. Prior to LC-MS/MS analysis samples were re-dissolved in 0.1% formic acid. For an experimental line-up see Figure 4.

### LC-MS/MS analysis

Tryptic peptides, from digestion of the gel separated secretome, were separated on a 15 cm capillary column (Zorbax SB300 C18, 0.075 mm ID) by reversed phase chromatography. Peptides were reconstituted in 0.1% formic acid, loaded onto the column in eluent A (0.1% formic acid) and separated with a linear gradient from 3% to 60% eluent B (84% acetonitrile in 0.1% formic acid) at a flow rate of 250–300 nL/min. Gradient lengths were 130 min and the LC system (Ettan MDLC, GE Healthcare Life Sciences, NJ, USA) was directly coupled in-line with a LTQ-FTICR instrument (Thermo Fisher Scientific) via a nano-electrospray source (Thermo Fisher Scientific). The source was operated at 1.4 kV, with no sheath gas flow and with the ion transfer tube at 200°C. The mass spectrometer was programmed for acquisition in a data dependent mode. The survey scans were acquired in the FTICR mass analyzer at a resolution of 50,000 at m/z 400 and covered the m/z range 300–2,000, the 7 most intense peaks in each full mass scan, with charge state ≥ 2 and intensity above a threshold of 100, were selected for fragmentation in the linear ion trap (LTQ) by collision induced dissociation (CID). CID fragmentation was performed with normalized collision energy of 35%, activation q = 0.25, activation time of 30 ms and three microscans were averaged. For all fragmentation events dynamic exclusion was enabled with a repeat count of 2, peaks selected for fragmentation more than twice within 30 s were excluded from selection (20 p.p.m. window) for 180 seconds, the maximum number of excluded peaks was 200.

### Protein identification and quantification

Raw data containing full-scan spectra acquired in profile mode and centroid MS/MS spectra, from the analysis of tryptic peptides, were processed using the MaxQuant software version 1.2.0.18 [65]. Default settings were used for feature extraction and first search for recalibration was performed against the human first search database provided with the software. The Andromeda search engine [66] integrated into the MaxQuant package was used for peptide identification and searches were performed against the IPI human database (version 3.68, 8,7061 sequences). Parameters for identification and quantification were set as follows: variable modification; oxidation of methionine and acetylation of the N-terminal, fixed modification; carbamidomethylation of cysteine, MS/MS tolerance 0.5 Da, peptide and protein FDR was set to 0.01 and for SILAC labeled samples the heavy label was set to arg6 and lys6. SILAC protein ratios are determined as the median of all peptide ratios assigned to the protein. For quantification a minimum peptide ratio count of two was set for each protein. To ensure that the Log2 values of the normalized protein H/L ratios followed a normal distribution and were centered around zero, histograms were plotted. Calculation of Significance B was done on the Log2 values of protein H/L ratios and intensities using the Perseus module (version 1.2.0.17) available in the MaxQuant environment. Threshold value for the significance B was set to 0.05 and the truncation was based on the Benjamini-Hochberg FDR.

### Comparison of datasets

MaxQuant result files (ProteinGroups.txt and peptides.txt), including information on identified proteins and peptides were imported into the ProteinCenter software version 3.8.2014 (Proxeon Bioinformatics, Odense, Denmark). Data were filtered so that each identified protein contained at least 2 unique peptides and identified proteins were clustered, based on peptide sharing, into groups of indistinguishable proteins. All protein identifications originating from the analysis of culture media from explant incubations (eight samples) were merged into one single data set and all protein identifications from monolayer cultures (five samples) were merged into a second data set. Comparison of the two datasets was performed in the Protein Center software. An additional comparison was made to 100 proteins identified from monolayer-cultured chondrocytes in a study by Polacek et al. [22]. These protein identifiers were manually added to the ProteinCenter software.

### Gene ontology analysis

The proteins that were differentially abundant when comparing the medial and lateral HM femoral condyle and the differentially abundant proteins when comparing the medial and lateral LM femoral condyle were put in a biological context by gene ontology (GO) analysis using Database for Annotation, Visualization and Integrated Discovery (DAVID) v6.7 [67,68]. The protein lists were uploaded to DAVID with official gene symbol as identifier and the gene ontology term Biological Process FAT (GOTERM_BP_FAT) was chosen for gene ontology analysis. Functional annotation chart was used for analyzing the functional annotation results. Genetic association database for disease was used in DAVID to analyze if the proteins common for both monolayer chondrocytes and explants were associated with any known disease.

### Verification of MS quantifications by Enzyme Linked Immunosorbent Assay (ELISA)

TIMP metallopeptidase inhibitor 1 (TIMP1) and matrix metalloproteinase-2 (MMP2) protein levels in the chondrocyte-conditioned media used for SILAC analysis were determined with ELISA according to the manufactures recommendations (R&D Systems, MN, USA). The samples were diluted 1:49 for the TIMP1 ELISA and used undiluted for the MMP2 ELISA. Optical density of each well was determined with an Infinite® F50 Absorbance microplate reader set to measure 450 nm with the wavelength correction set to 540 nm (TECAN, Männedorf, Switzerland). Measurement data and protein amounts were calculated using the Magellan™ V6.6 software (TECAN).

## Competing interests

The authors declare that they have no competing interests.

## Authors’ contributions

JS and UR designed the study, carried out the cell cultures, performed and analyzed the proteomic analysis together with the statistic analysis and drafted the manuscript. ES and JK participated in the study design and helped to draft the manuscript. AL conceived the study, analyzed the proteomic data and participated in drafting the manuscript. All authors have read and approved the final manuscript.

## Supplementary Material

Additional file 1: Table S1 Proteins identified in cellmedium from explant cultures of two OA patient samples (Patient 1 and Patient 2). Cellmedia collected at day 2 and 10 were collected and analysed in duplicates. Click here for file

Additional file 2: Table S2 Proteins identified in cellmedium from monolayer cultures of five OA patient samples. Click here for file

Additional file 3: Table S3 Proteins common for the combined OA explant seretome and the combined high Mankin scored monolayer secretomes. Click here for file

Additional file 4: Table S4 Gene Ontology analysis. Click here for file

Additional file 5: Table S5 Proteins identified in cellmedium from monolayer cultured chondrocytes of one LM-scored patient sample. Duplicate samples were individually SILAC labelled and processed in parallel. LM Exp I = Low Mankin label experiment one, LM Exp II = Low Mankin label experiment two. Click here for file

Additional file 6: Table S6 All medially/latterally regulated proteins in the HM secretomes. Silac protein ratio>1= Femur medial abundance. Silac protein ratio<1= Femur lateral abundance. HM=High Mankin scored individual. NR= No Ratio. Click here for file

Additional file 7: Table S7 Up-regulated proteins in the LM femur lateral secretome. LM I=Low Mankin scored individual experiment one. LM II=Low Mankin scored individual experiment two. HM.1-5=High Mankin scored individual 1-5. NR=No Ratio. Click here for file

Additional file 8: Table S8 Proteins uniquely found in the LM secretome. Click here for file
